# Toward fully automated UED operation using two-stage machine learning model

**DOI:** 10.1038/s41598-022-08260-7

**Published:** 2022-03-10

**Authors:** Zhe Zhang, Xi Yang, Xiaobiao Huang, Timur Shaftan, Victor Smaluk, Minghao Song, Weishi Wan, Lijun Wu, Yimei Zhu

**Affiliations:** 1 SLAC National Accelerator Laboratory, Menlo Park, CA 94025 USA; 2 National Synchrotron Light Source II, Brookhaven National Laboratory, Upton, NY 11973 USA; 3 School of Physical Science and Technology, ShanghaiTech University, Shanghai, 201210 China; 4 Condensed Matter Physics and Materials Science Division, Brookhaven National Laboratory, Upton, NY 11973 USA

**Keywords:** Materials science, Mathematics and computing, Physics

## Abstract

To demonstrate the feasibility of automating UED operation and diagnosing the machine performance in real time, a two-stage machine learning (ML) model based on self-consistent start-to-end simulations has been implemented. This model will not only provide the machine parameters with adequate precision, toward the full automation of the UED instrument, but also make real-time electron beam information available as single-shot nondestructive diagnostics. Furthermore, based on a deep understanding of the root connection between the electron beam properties and the features of Bragg-diffraction patterns, we have applied the hidden symmetry as model constraints, successfully improving the accuracy of energy spread prediction by a factor of five and making the beam divergence prediction two times faster. The capability enabled by the global optimization via ML provides us with better opportunities for discoveries using near-parallel, bright, and ultrafast electron beams for single-shot imaging. It also enables directly visualizing the dynamics of defects and nanostructured materials, which is impossible using present electron-beam technologies.

## Introduction

To take full advantage of mega-electron-volt (MeV) electrons with large scattering power with matter and mitigated space charge effect, and easy to focus with simultaneously high spatial and temporal resolution toward ultrafast electron diffraction (UED) and microscopy (UEM), we show that one can apply ML to optimize the UED/UEM instrument, with the capability of providing a near-parallel, spectrally bright, and ultrafast electron beam for high-charge single-shot imaging. Besides, a fully automated UED instrument can serve different types of user experiments with the maximum efficiency. Owing to the inherent RF-related out of sync and fluctuations in UED/UEM instruments, obtaining accurate electron beam properties (e.g., emittance, energy spread, spatial-pointing jitter, and shot-to-shot energy fluctuation) is the key to success. This demands real-time nondestructive diagnostics for each electron pulse. A self-consistent start-to-end simulation from the gun to the detector, implemented in our early study^1–3^, will be used to demonstrate the feasibility of fully automated UED operation and real-time diagnosis of the machine performance. The start-to-end simulation is performed through particle tracking from the gun to the sample via the General Particle Tracer (GPT) code^4^ and wave-like electrons being diffracted by the sample to the detector via the Electron Diffraction Pattern (EDP) code^5–8^. The GPT code takes the 3D space charge and stochastic-scattering effects into full consideration and has been bench-marked with several well-established simulation codes, such as ASTRA and PARMELA^4,9–12^. The EDP code is based on the Bloch wave method, which takes the dynamical effects of electron diffraction into account and has been successfully used to quantitatively determine the crystal structures and charge distributions^5–8^. For a proof-of-concept study, the GPT model from the gun to the sample can be considered a true representation of the UED instrument and the EDP simulations from the sample to the detector can reproduce almost every UED experiment. In GPT simulation, there are several key machine parameters, which are essential and necessary to determine the electron beam properties for each individual simulation run. They must be varied in the proper ranges, which are sufficiently large to cover the complete parameter space of a UED instrument. As a result, the GPT model can nearly reproduce all potential experimental configurations.

For a proof-of-concept test, results of start-to-end simulation are generated to build an ML model. The model takes Bragg-diffraction (BD) patterns recorded by the detector as the input and make predictions of the machine parameters, with the goal of enabling fully automated UED operation. The potential benefits are: (1) the ML model can be applied to tune up the beamline in real time; (2) the electron beam properties can be optimized based on the special requirements of each individual experiment, e.g., the high-charge single-shot mode and the low-charge accumulation mode^3^; (3) automating the UED instrument can greatly improve the efficiency of the user experiments via reducing the time required to set up the accelerator; (4) this is one step closer toward our ultimate goal of achieving high spatiotemporal resolution of tens of nm∙ps in the UED/UEM experiment. The combination of mitigated space charge effect via the MeV electron beam energy, ultrahigh bright photocathode RF gun, and the real-time global optimization of electron beam properties over a given complete parameter set via ML could avoid the local minimum, and therefore, allow maximum electrons to be packed in the most critical dimensions, i.e., the divergence and the energy spread, which affect the UED/UEM resolution for single shot analysis^3^. The capability enabled by the ML model provides us with the opportunities for new sciences using a near-parallel (both x and y divergences of 18 µrad instead of 140 µrad in our early study^1^), ultrafast, and bright electron beam for single-shot imaging, for directly visualizing the dynamics of defects and nanostructured materials, or even recording molecular movie^13–26^. Such a UED/UEM instrument not only can be easily accessed by a university-scale laboratory, but also can be a valuable complement to the cutting-edge XFEL facilities in ultrafast science and technology.

## Results

### Selection of the ML model configuration

**Figure 1:**
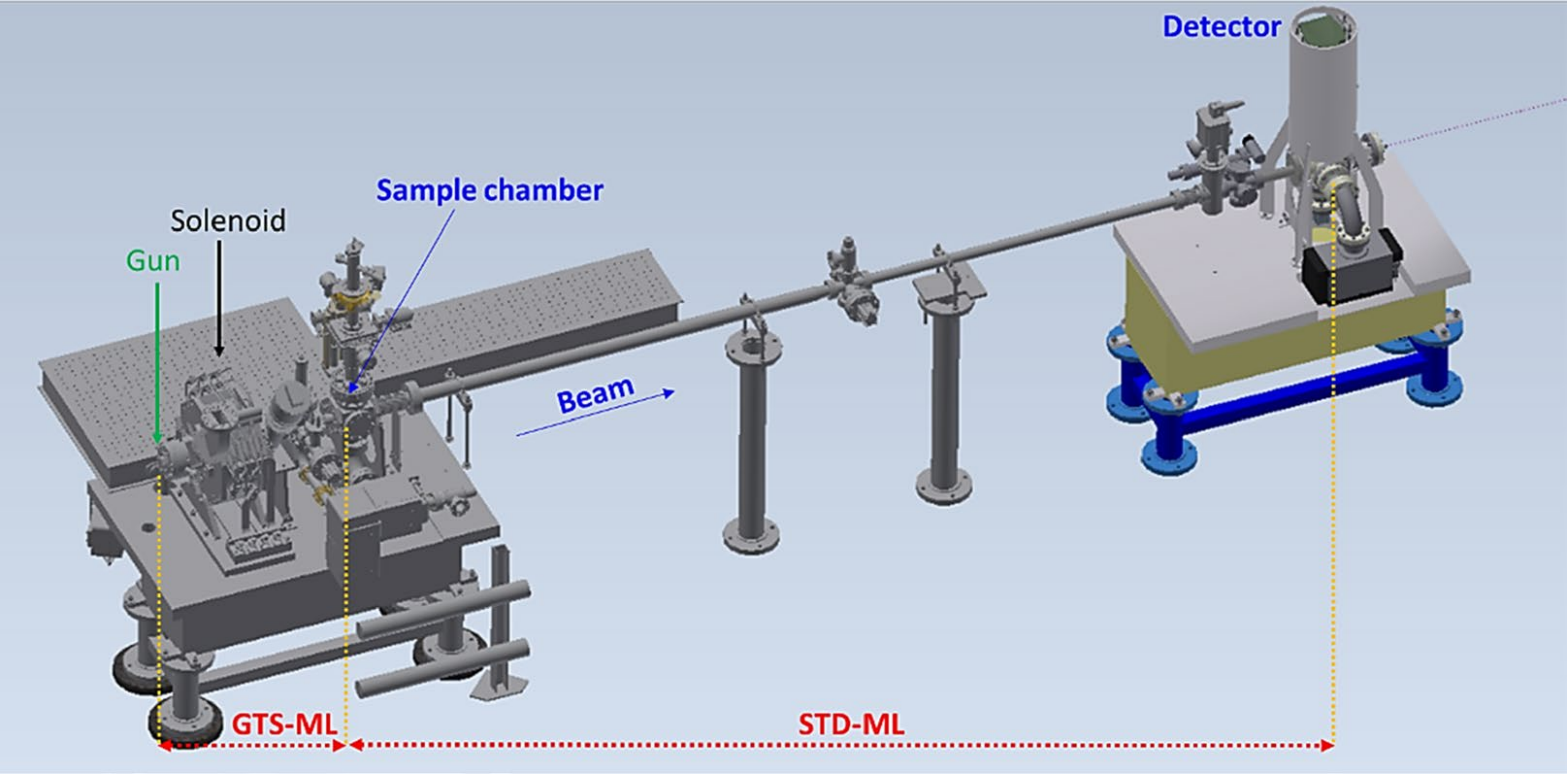
Schematic layout of the UED instrument with the positions of the gun, solenoid magnet, UED sample chamber and detector.

**Figure 2:**
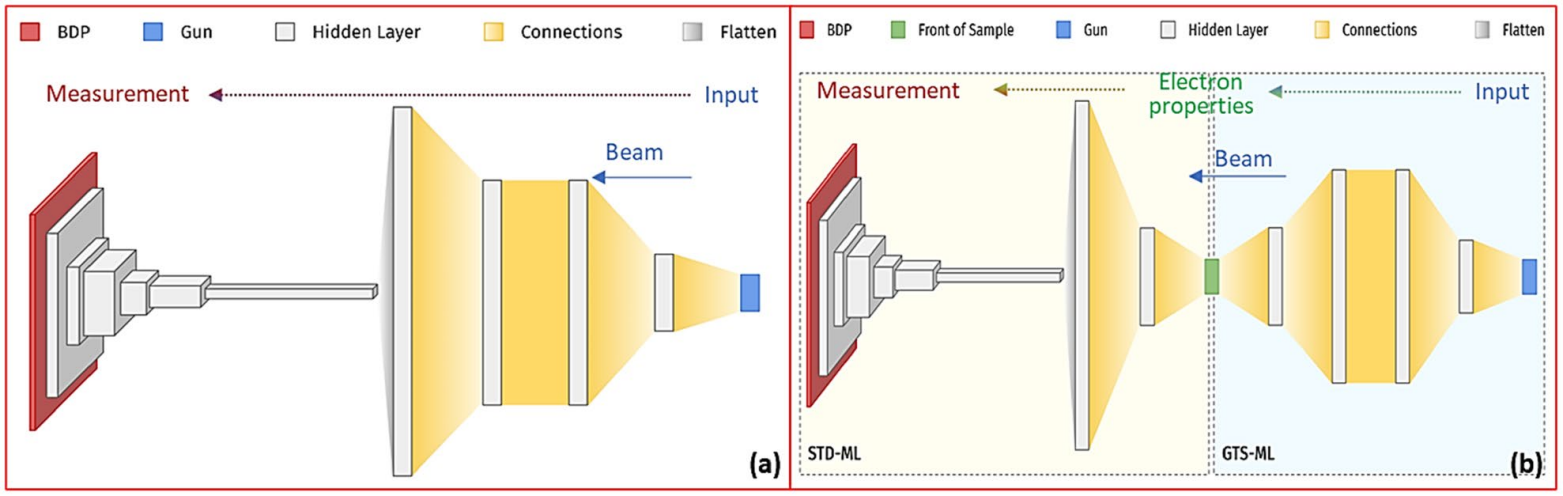
Schematic layout of the ML model regarding: (**a**) Option 1, one NN covers the entire UED instrument, from the gun to the detector. (**b**) Option 2, the UED beamline is represented by two sequential NNs, from the gun to the sample and from the sample to the detector, respectively. Beam goes from right to left.

A schematic layout of a typical UED instrument is shown in Fig. 1. The UED instrument has several main components: the photocathode RF gun, the solenoid magnet, the UED sample chamber, and the detector^27,28^. To build the ML model, an optimal configuration was chosen from several options. The first option is one neural network (NN) based ML model that covers the entire UED instrument, from the gun to the detector, as shown in Fig. 2a; the second one takes a two-stage approach to build the ML model, which comprises two sequential NNs, as shown in Fig. 2b. The first NN starts from the gun to the sample, named GTS-ML, and the second one starts from the sample to the detector, named STD-ML.

Among the two different types of ML configurations, the two-stage approach is preferred for the following reasons. For the first option, as one NN covers the entire beamline, the potential applications are quite limited. The BD patterns and machine parameters are the input data and output predictions of this ML model, respectively. It only can be used to automate the setup of a UED instrument as no intermediate information is provided. Alternatively, in the second option, by separating the beamline into two parts^29^, from the gun to the sample and from the sample to the detector, shown as the schematic layout of the UED beamline in Fig. 1 and the schematic layout of the NN model in Fig. 2b, we gain some advantages. This two-stage approach has much broader applications because of the availability of the electron beam properties. The two sequential NNs, GTS-ML (sample independent) and STD-ML (accelerator independent)^30^, are mutually independent. As a result, the frequency of updating the two-stage model can be lower; instead, one only needs to update the stage model with changes. Since an ML model is purposely built to solve the complex inverse problem, in our case, based on the BD patterns recorded by the detector as the input data, the two-stage ML model will ultimately make the predictions of the machine parameters, which will be used to automate the setup of a UED instrument. Furthermore, the predictions by the second NN (STD-ML), the electron beam properties at the sample, will not only provide the input to the first NN (GTS-ML) to enable the full automation of the UED instrument, but also make the real-time electron beam information available for data binning, filtering, correcting, and integrating, which enables single-shot noninvasive diagnostics for the UED instrument with high repetition rate and high fidelity. In a summary, one can apply the two-stage ML model not only to automate the UED instrument, but also to achieve real-time single-shot electron beam diagnostics while the experiments are ongoing. It is more important that the second option is built on the success of our early studies^30^. By adopting the two-stage approach, the demonstrated STD-ML model with a few new developments can be applied as the second NN.

In this report, we mainly focus on demonstrating the feasibility of the GTS-ML model and characterizing its performance. First, we perform the simulation study using the GPT code. A GTS-ML model is subsequently built based on the simulation results. We show that not only the predicted machine parameters meet the required precision, but also the prediction is extremely fast, taking less than 0.5 µs in the batch mode. In addition, to improve the STD-ML model, we have applied the hidden correlation between the electron beam properties and the features of a BD pattern as the model constraints, successfully improving the accuracy of the energy spread prediction by a factor of five and making the beam divergence prediction two times faster.

## Feasibility study of the GTS-Ml model

### Building a model

To demonstrate the feasibility of the GTS-ML model, GPT simulations from the gun to the sample provide the required training datasets to build the GTS-ML model. The input to the GPT simulation are several key machine parameters, including the spot size, x-jitter and y-jitter of the center position of an electron bunch emitted by the photocathode, the amplitude and phase of the RF waveform, and the current of the solenoid magnet. The charge distribution of an electron beam emitted by the photocathode is primarily determined by the characteristics of the laser pulse since the photoelectron emission from the cathode is prompt with respect to the laser light^31^. The output of the GPT simulations are the electron beam properties, including the x and y divergences, x and y spatial positions, energy, and energy spread, at the sample. The jitters of the sample can be neglected due to the following reasons: (1) drift, fluctuation, and vibration of a sample do not change the BD pattern since the electron diffraction is in Fourier space; (2) although mechanical expansion and compression of a sample could alter the BD pattern, such effects are usually much smaller compared to the peak broadening caused by the energy spread and divergences of the electron beam. The electron beam properties and the machine parameters are the input and output parameters of the GTS-ML model, respectively. For the ML models in both stages, each training data set is divided into three subsets: the training set, the validation set, and the test set.

The reasons to choose these six machine parameters as variables for each individual run of the GPT simulation while all other parameters are fixed are: (1) they are the main parameters determining the electron beam dynamics^9–12,31^; (2) they are highly correlated with the output of the simulation (i.e., the electron beam properties at the sample); (3) their settings are absolutely necessary in determining the electron beam properties at the sample, which are unique and specific for different type of the UED experiments (i.e., the high charge single-shot and low charge accumulation modes)^3^; (4) they are often fluctuant and, therefore, need to be constantly tuned during an experiment. Other parameters, e.g., the pulse duration of a photocathode drive laser system and the standard charge of a UED instrument, are fixed to 250 fs in full width half maximum and 0.5 pC, respectively, as they are comparably more stable in the duration of an experiment. Compared to the inherent RF-related out of sync and fluctuations^1–3^, the jitters caused by the drive laser properties and photon emission processes are often negligible (at least one order of magnitude smaller)^31,32^.

To take the 3D space charge effect into a proper consideration, we choose spacecharge3Dmesh, which is a mesh-based routine, as the 3D space-charge model. It is not only fast (computing time scales almost linearly with the number of particles) but also reliable for a 0.5 pC short electron bunch with low energy spread^9^. The time over which the particles travel from the gun to the sample is 4.18 ns and the step size is set to 5 ps. The number of particles is chosen to be 10,000, which is a good compromise between accuracy and computing speed. The GPT simulation has been bench-marked with our early study result of the influence of the focusing magnets on the beam size^1^.

To have the full coverage of the parameter space of a UED instrument, we build a table based on the Latin hypercube sampling scheme^30,33^. The Latin hypercube sampling method provides an efficient coverage of the parameter space with the maximum randomness and the highest density of the population. Each set of the machine parameters, which corresponds to a row in the table and determines an individual GPT simulation run, has a total number of 10,000 values, hence, 10,000 $\times$ 6 data points. The ranges of variations for these six machine parameters are determined by the experimental setup of the UED beamline^1,2^. Each GPT simulation run provides a set of the electron beam properties at the sample, including x and y divergences, x and y spatial positions, energy, and energy spread.

To build the GTS-ML model, the results of the GPT simulations form a training dataset. To be specific, for each training data set, the input data are the electron properties (p1, p2, …, p6) at the sample, including x and y spatial positions (*avg*(*x*) and *avg*(*y*)), x and y divergences (*std*(*x*′) and *std*(*y*′)), energy spread and energy (∆*E/E* and $\gamma$), and the output are the six machine parameters (v1, v2, …, v6), x-jitter (*x*_*off*_), y-jitter (*y*_*off*_) and spot size (*σ*_*r*_) of the electron bunch emitted at the photocathode, amplitude jitter (*∆V*_*RF*_* / V*_*RF*_) and gun phase (*θ*_*gun*_) of the RF waveform, and the normalized current of the solenoid magnet (*I / I*_*max*_), as shown in Table 1. In GPT code. The beam energy is represented by the Lorentz factor $\gamma$.

**Table 1:** The training variables (data label), *v*_1_, *v*_2_, *v*_3_, *v*_4_, *v*_5_, and *v*_6_, with respect to the electron properties (input data), *p*_1_, *p*_2_, *p*_3_, *p*_4_, *p*_5_ and *p*_6_, are listed, including the upper maximum and lower minimum limits for all parameters and their physical definitions.

Variable	Min	Max	Unit	Formula	Description	Beam properties	Formula	Description
*v*_*1*_	0	5	μm	*x*_*off*_	x-jitter	*p*_*1*_	avg(*x*)	x-position
*v*_*2*_	0	5	μm	*y*_*off*_	y-jitter	*p*_*2*_	avg(*y*)	y-position
*v*_*3*_	30	220	μm	σ_*r*_	Spot size	*p*_*3*_	std(*x'*)	x-divergence
*v*_*4*_	− 0.025	0.025		*∆V*_*RF*_*/ V*_*RF*_	RF amplitude jitter	*p*_*4*_	std(*y'*)	y-divergence
*v*_*5*_	5	60	degree	*θ*_*gun*_	RF phase	*p*_*5*_	*∆G*	Energy spread
*v*_*6*_	0	0.4		*I / I*_*max*_	Solenoid factor	*p*_*6*_	*G*	Energy

Compared to the RF synchronization (*θ*_*gun*_) and fluctuations (*∆V*_*RF*_* / V*_*RF*_), the laser pulse duration (*σ*_*t*_) and total charge (*Q*_*tot*_) are more stable and fixed in the GPT simulation.

### Optimizing the model-architecture

**Figure 3:**
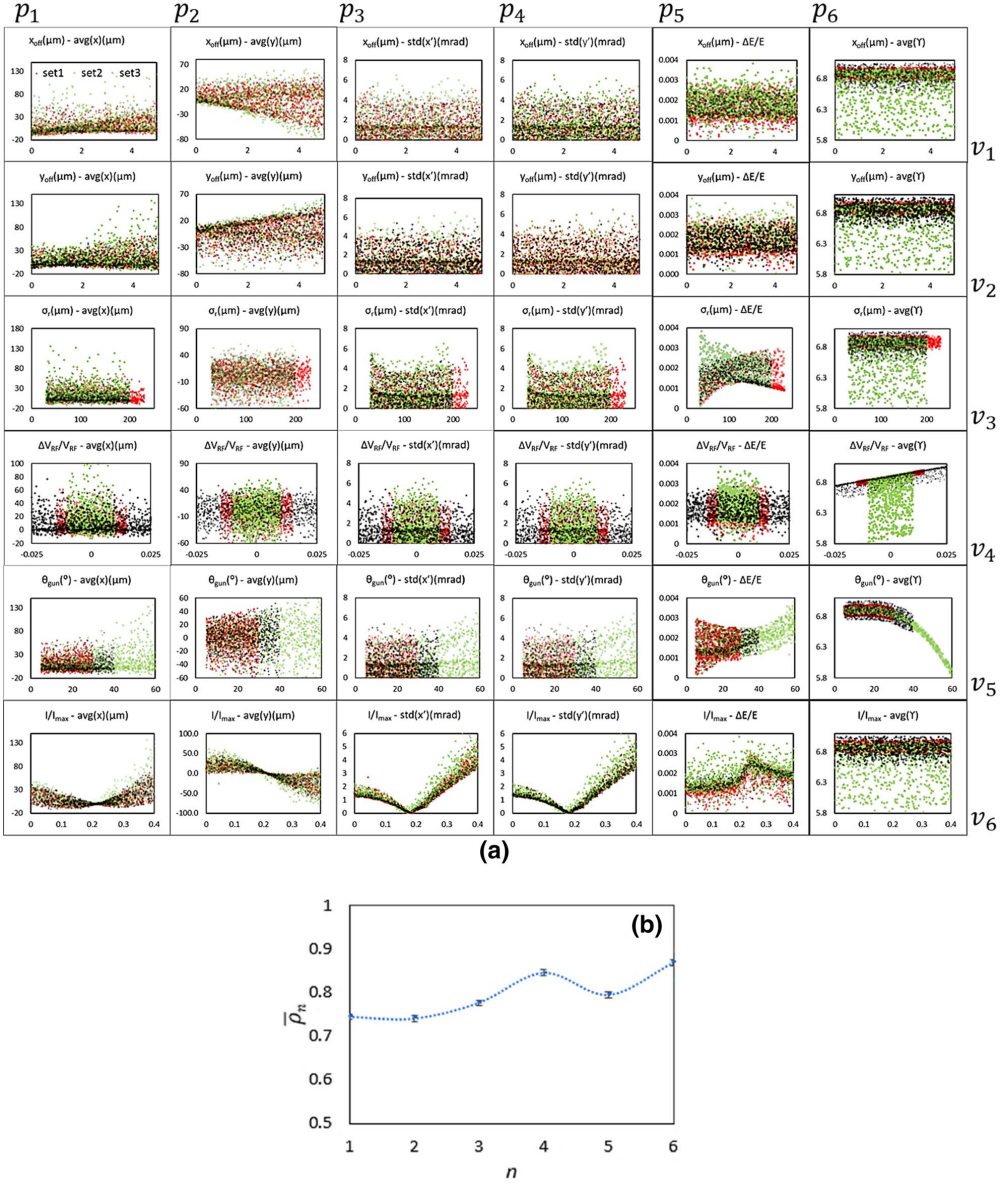
(**a**) To achieve a high accuracy, three parameters (*v*_*3*_ (*σ*_*r*_), *v*_*4*_ (*∆V*_*RF*_*/V*_*RF*_) and *v*_*5*_ (*θ*_*gun*_)) that have the most significant impact on the space charge effect, are varied in GPT simulations to generate three different training datasets (named set #1, #2, and #3). For these three training data sets, the correlations between the input and output parameters are plotted. Set #1, #2 and #3 are plotted as the red, green, and black dots, respectively. x-axes are the data labels of the 6 machine parameters (v1, v2, …, v6), which correspond to row 1, 2, 3, 4, 5, and 6, respectively; y-axes are the electron beam properties (p1, p2, …, p6), which correspond to column 1, 2, 3, 4, 5, and 6, respectively. Also, the tile of each subplot provides the information of the plotted machine parameter as the x-axis and electron beam property as the y-axis. (**b**) Mean correlation ($\overline{\rho }_{n}$) *vs* index (*n*) of machine parameters. Error is estimated via the RMS deviation of all 3 datasets.

The relationships between the input and output parameters of the training results are shown as the correlation plots in Fig. 3a. The x-axes are the data labels of those six machine parameters (*v*_*1*_, *v*_*2*_, …, *v*_*6*_), which determine the GPT simulation conditions, and the y-axes are the electron beam properties (*p*_*1*_, *p*_*2*_, …, *p*_*6*_), which are the output of the GPT simulation. Mean correlation ($\overline{\rho }_{n}$) *vs* index (*n*) of the machine parameters is plotted in Fig. 3b. The mean correlation is calculated via Eq. (1), where $\rho_{n,j}$ is the correlation between *v*_*n*_ and *p*_*j*_ (*n*, *j* = 1, 2, …, 6) and *M* is the size of the training dataset.

1 $$\overline{\rho }_{n} = \mathop \sum \limits_{j = 1}^{6} \rho_{n,j} /6,\quad with\quad \rho_{n,j} = \mathop \sum \limits_{i = 1}^{M} v_{n}^{\left( i \right)} \cdot p_{j}^{\left( i \right)} /\sqrt {\mathop \sum \limits_{i = 1}^{M} v_{n}^{\left( i \right)2} \cdot \mathop \sum \limits_{i = 1}^{M} p_{j}^{\left( i \right)2} }$$

**Figure 4:**
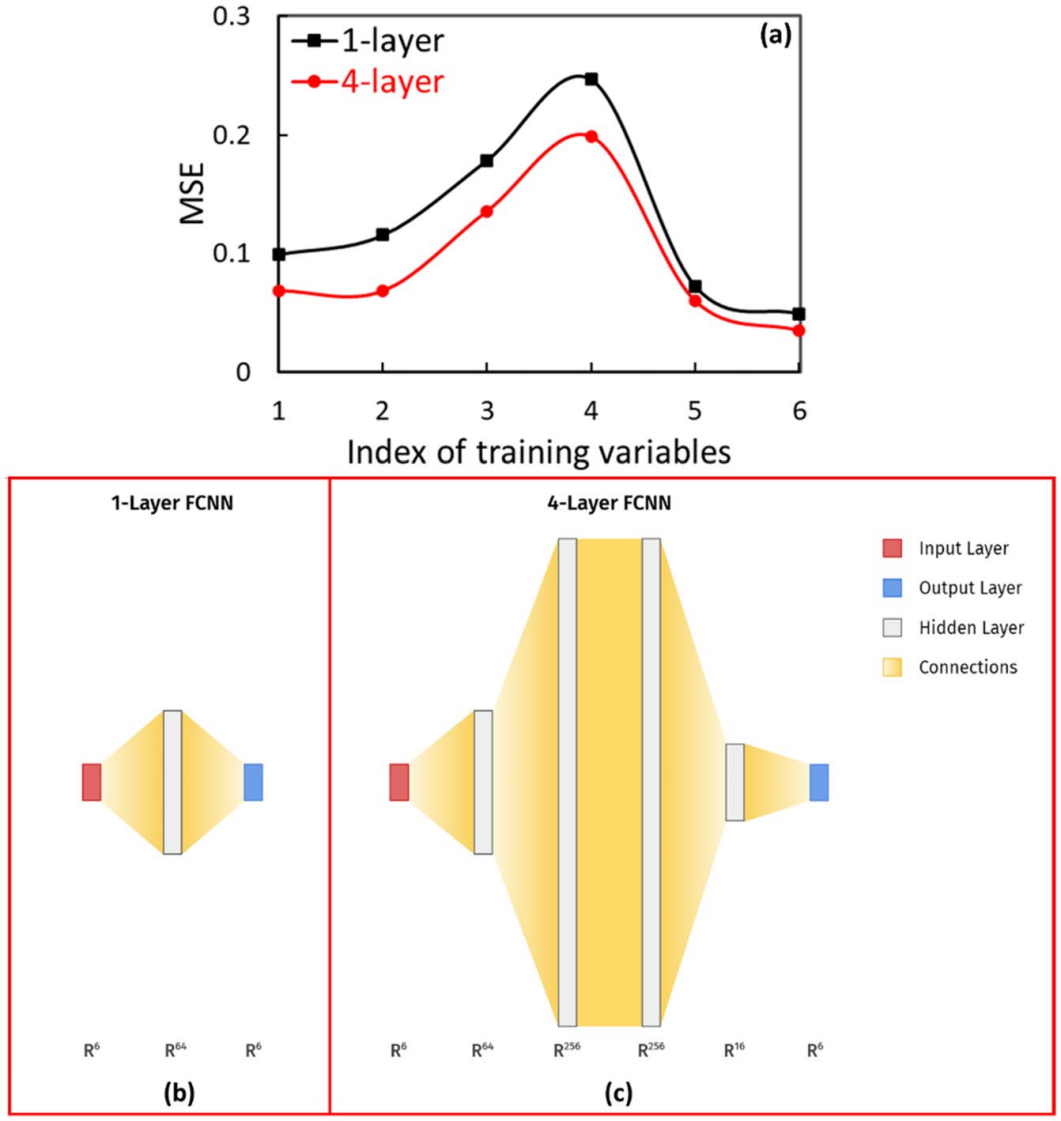
(**a**) For the same training data set, the results of 1-layer (black) and four-layer (red) ML models are plotted as a function of the training variable index. Schematic of 1-layer (**b**) and 4-layer (**c**) models. FCNN represents fully connected neural network. The number of nodes for each layer in these two models are shown below the model schematic plot.

Since the input and output parameters are highly correlated (i.e., *v*_*6*_ (0.87)), we expect the training of the GTS-ML model to be successful. The results are quite reasonable except—the predicted precision of *v*_*4*_ (*∆V*_*RF*_*/V*_*RF*_) is significantly worse than we initially expected, having a mean square error (MSE) of as large as 0.24 via a simple 1-layer ML model, shown as the black curve in Fig. 4a. The reason for the poor performance could be the space charge effect that makes the relationships between the machine parameters and the electron beam properties at the sample very nonlinear; therefore, the 1-layer ML model (Fig. 4b) is too simple to reproduce such a complicated nonlinear dynamic process. To take the space-charge induced nonlinear effect into a proper consideration, a 4-layer ML model (Fig. 4c) has been implemented. The number of trainable parameters is 838 and 87,094, for the 1-layer model and 4-layer model, respectively. For the same training data set, the 4-layer ML model significantly improves the accuracy of the model prediction, by about 50% in terms of the MSEs of untrained data sets for all six machine parameters, shown as the red curve in Fig. 4a. On the other hand, the complexity of the 4-layer model is sufficient to fit the data, while overfitting was under control with the early stopping strategy^34^ on the 10,000-point training dataset. As the result, this 4-layer NN configuration will be chosen to build the GTS-ML model.

### Defining machine parameter-space

**Figure 5:**
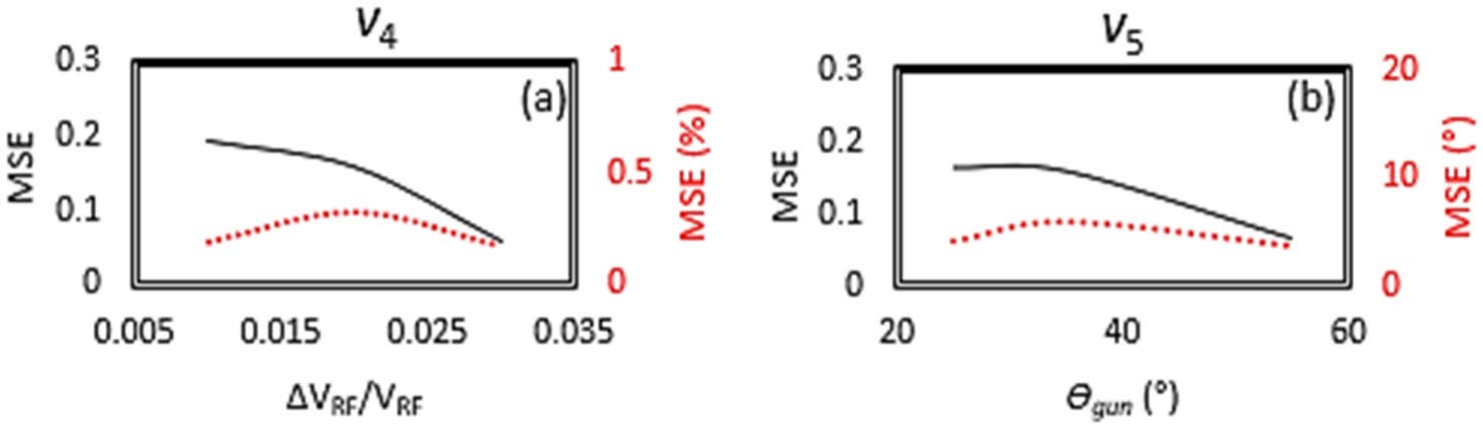
MSE *vs* range of the variation of *v*_4_ (**a**) and *v*_5_ (**b**) are plotted as the primary (black) and secondary (red) y-axes, which correspond to before and after the unit conversion, respectively.

Since *v*_*3*_ (*σ*_*r*_), *v*_*4*_ (*∆V*_*RF*_*/V*_*RF*_) and *v*_*5*_ (*θ*_*gun*_) are the machine parameters, with the most significant impact on the space charge effect, they are the most challenging ones to be predicted with high fidelity. To achieve the best possible accuracy, these three parameters are varied in GPT simulations to generate three different training datasets (named set #1, #2, and #3) for comparison, shown in Fig. 3a as red, green, and black dots, respectively. The ranges of the variations of those six training variables (*v*_1_, *v*_2_, *v*_3_, *v*_4_, *v*_5_, and *v*_6_) and their MSE via the ML model are listed in Table 2 for data set #1, #2, and #3 with some details. Since both *v*_*4*_ (*∆V*_*RF*_*/V*_*RF*_) and *v*_*5*_ (*θ*_*gun*_) affect the electron beam energy, *v*_*4*_ and *v*_*5*_ have been deliberately varied much more in set #3 (± 2.5%, as black dots in row 4 of Fig. 3a) and set #2 (5°–60°, as green dots in row 5 of Fig. 3a), respectively. As the result, the optimal ranges of variations are determined and their corresponding MSEs are listed in the Summary columns of Table 2. With the improved 4-layer deep NN ML model, the ranges of variations of data set #2 generate the highest precisions with the MSE reduction from 30.0 µm to 22.8 µm for the spot size of the electron beam, from 0.006 to 0.003 for the RF amplitude jitter, and from 5.9° to 3.7° for the gun phase. It is likely that the residual MSEs of those six machine parameters are limited by the space charge induced nonlinear effect, since the dependence of the MSE on the range of variation is not so strong. The MSEs of data set #1 and set #2 are quite similar, especially for *v*_*4*_ and *v*_*5*_ after unit conversion, shown as the secondary y-axis (red) in Fig. 5a and b respectively. Also, any increase of the size of a training dataset beyond 10 k doesn’t help in improving the fidelity of the prediction any further.

**Table 2:** The ranges of variations of those six training variables (*v*_1_, *v*_2_, *v*_3_, *v*_4_, *v*_5_, and *v*_6_), including the upper maximum and lower minimum limits, and MSEs regarding the entire dataset, including before and after unit conversion, are listed for those three data set #1, #2, and #3.

Variable	Formula	Description	Unit	Set 1 (10 k)			Set 2 (20 k)			Set 3 (100 k)			Summary		
				Min	Max	MSE	Min	Max	MSE	Min	Max	MSE	Min	Max	MSE/target
*v*_*1*_	*x*_*off*_	x-jitter	μm	0	5	0.0731/0.3655 μm	0	5	0.0766/0.3830 μm	0	5	0.0807/0.4035 μm	0	5	0.38 μm/0.50 μm
*v*_*2*_	*y*_*off*_	y-jitter	μm	0	5	0.0738/0.3690 μm	0	5	0.0707/0.3535 μm	0	5	0.0791/0.3955 μm	0	5	0.35 μm/0.50 μm
*v*_*3*_	σ_*r*_	spot size	μm	30	220	0.1479/28.101 μm	30	200	0.1343/22.831 μm	30	200	0.1762/29.951 μm	30	200	22.8 μm/30 μm
*v*_*4*_	*∆V*_*RF*_*/ V*_*RF*_	RF amplitude jitter		− 0.015	0.015	0.0595/0.002	− 0.01	0.01	0.1650/0.003	− 0.025	0.025	0.1124/0.006	− 0.01	0.01	0.003/0.002
*v*_*5*_	*θ*_*gun*_	RF phase	degree	5	30	0.1651/4.128°	5	60	0.0677/3.724°	5	40	0.1682/5.880°	5	60	3.7°/3°
*v*_*6*_	*I*/*I*_*max*_	Solenoid factor		0	0.4	0.0307/3.07%	0	0.4	0.0269/2.69%	0	0.4	0.02912/2.91%	0	0.4	2.7%/3%

The optimal ranges of variations, corresponding MSEs and targeted precisions of those six machine parameters are listed in the summary.

To establish a deterministic one-to-one map between the predicted machine parameter by the ML model and the experimental setting of the UED instrument, the accuracy of the six machine parameters must match well with the precision of the UED instrument. The targeted precisions of the machine parameters are listed in Summary of Table 2. As expected, we have achieved the reasonably good agreement for the 200 untrained machine parameters via the trained GTS-ML model, fulfilling the precision demanded by the automation of the UED instrument. It is evident that the output of the GTS-ML model could provide the machine parameters with the adequate precision, for the fully automated UED operation. Afterwards, a scan of each individual machine parameter around the model predicted optimal setting can further reduce the MSE in cases that higher precision of the machine parameters is required for more challenging experiments, i.e., those with the minimum energy spread and divergence of the electron beam. The ML model will predict *v*_3_ and *v*_5_ with the minimum *σ*_*r*_ and *θ*_*gun*_, shown as the ‘*σ*_*r*_—∆*E/E*’ (*v*_3_ – *p*_5_) and ‘*θ*_*gun*_—∆*E/E*’ (*v*_5_ – *p*_5_) plots in Fig. 3a, for the minimum energy spread, and particularly set *v*_6_ with an optimal value of 0.18 for the minimum x and y divergences, shown as ‘(*I/I*_*max*_—*std*(*x*′)’ (*v*_6_ – *p*_3_) and ‘(*I/I*_*max*_—*std*(*y*′)’ (*v*_6_ – *p*_4_) plots. Fine tuning via scanning *σ*_*r*_, *θ*_*gun*_, *I / I*_*max*_, and other parameters in-situ can further optimize the energy spread and divergence.

## Improving the STD-Ml model

The STD-ML model has been successfully demonstrated in our early studies^30^. It can map every BD pattern to the corresponding underlying electron beam properties (*x* and *y* spatial positions, *x* and *y* angular divergence, energy, and energy spread). The BD patterns are labeled with the electron beam properties used to generate such patterns and are the input parameters to the ML model. To meet the continuous output variable requirement, a LeNet-style neural network architecture with a linear output activation function is used to build the ML model^35^. We demonstrate that all electron properties except energy and energy spread have the generalization errors on the order of 0.01 with respect to the normalized range of [0, 1] for the training variables. The prediction only takes less than 0.5 µs in batch mode^30^.

Based on our understanding of the connections between the electron beam properties and the features of a BD pattern^2,3^, we further improve the predictability of the most challenging electron beam parameters, energy spread and divergences, resulting in better precision and higher speed. This improvement is mainly driven by the need for real-time high-precision diagnostics. For a BD pattern, the transverse and radial peak broadenings are mainly caused by the *x* and *y* divergences and the energy spread of the electron beam, respectively, while peak positions are intrinsically correlated to the spatial positions of the beam. To improve the STD-ML model, we implement a two-step approach, which applies the brightest peak analysis (BPA) and polar coordinate transformation (PCT) in sequence. The two steps target on predicting the divergence and the energy spread, respectively. As a result, the new procedure for implementing the STD-ML model is: 1) apply BPA to predict the x and y positions and divergences; 2) center the BD pattern to the image by removing the x and y position jitters; 3) perform PCT; 4) train an ML model with the PCT BD patterns, which only predicts the energy spread and energy. Application of the new procedure results in a five times more accurate prediction of the energy spread.

### PCT for a better and faster energy spread prediction

To take advantage of the fact that the electron beam energy spread only causes peak broadening in the radial direction, an early version of PCT (named PCT-*v*1) was applied to resample a BD pattern, extract the pixels in the ring, and cast into a rectangular image with a more than four times size reduction^30^. However, this approach suffers a significant reduction in the prediction accuracy. To overcome such a limitation, a modified PCT (named PCT-*v*2), one that is relative to the center of a BD pattern instead of the center of an image, has been implemented. Compared to PCT-*v*1, PCT-*v*2 reduces the MSE of the training loss by more than a factor of five.

The detailed procedure of PCT-*v*2 is as follows: (1) obtain x and y position jitters for each BD pattern via the method described in section III-b; (2) remove x and y position jitters from each BD pattern; (3) apply PCT-*v*2 to those BD patterns; (4) train an ML model with the PCT-BD patterns as the input, which only predicts the energy spread and the energy.

**Figure 6:**
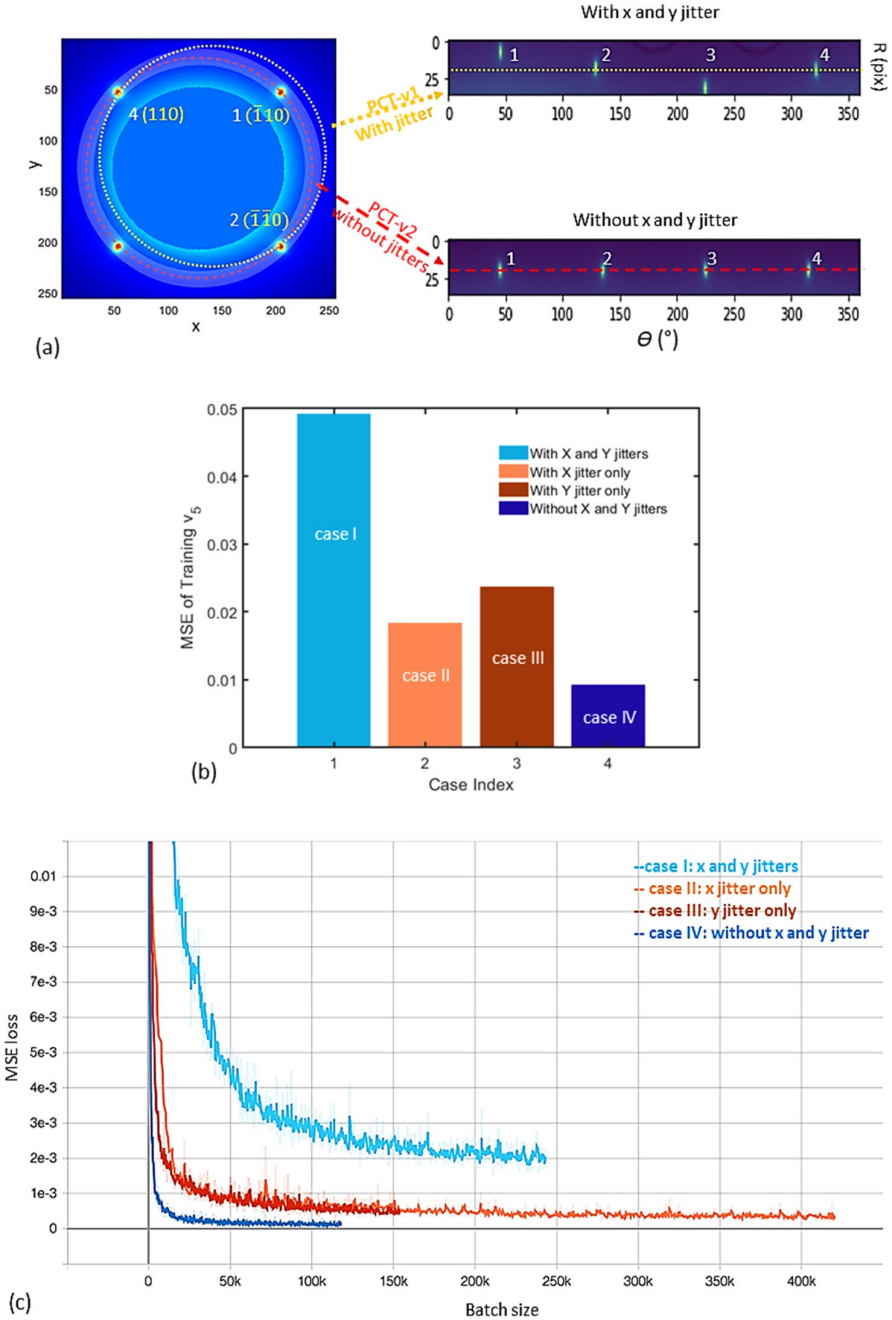
(**a**) An example of simulated BD patterns of SrTiO_3_ single crystal along [001] direction in the dataset with the RMS energy spread of (1.3·10^–2^) is plotted as the left graph. The image has a size of 256·256 pixels, with an angular resolution of 80 pixel/mrad in the EDP simulation. The x and y spatial pointing jitters in RMS are 36 μrad. For each image, the view angles are the same in *x* and *y* directions: ± 1.6 mrad. Miller indexes of those four selected Bragg peaks, 1, 2, 3, and 4, are ($\overline{1}10$), ($\overline{1}\overline{1}0)$, (1 $\overline{1}$ 0), and (110), respectively. PCT1 is to perform PCT regarding the image center with x and y position jitters; the result is shown as the top right graph. The yellow dotted circle in the BD pattern is equivalent to the yellow dotted line in the top right graph. After PCT-v1, peaks 1, 2, 3, and 4 are misaligned in the radial direction *R*. PCT-v2 is to perform PCT relative to the BD-pattern center (after the removal of x and y jitters) and the result is shown as the bottom right graph. The red dashed circle in the BD pattern is equivalent to the red dashed line in the bottom right graph; peaks 1, 2, 3, and 4 are well aligned in *R*. The cutoff radius in the middle of the BD pattern of Fig. 6a represents the hole in the middle of the mirror which is part of the detector system. The hole allows the background noises generated by the core of the non-interacted electron beam and by the dark current to pass through the mirror to the beam dump. (**b**) For the 200 untrained images, the corresponding MSEs of case- I, II, III, and IV are plotted as the light blue, light orange, dark orange, and dark blue bars, respectively. (**c**) Training loss vs batch size are plotted as the light blue, light orange, dark orange, and dark blue curves regarding to case I, II, III, and IV, respectively. The smoothed training losses are plotted on top of the raw data (the translucent curves).

A set of ten thousand images with the size of $256 \cdot 256$ pixels are generated based on the initial population set by the Latin hypercube sampling^33^. The labels of those images are normalized to the range of zero to one for the purpose of standardizing the training process. One example of the input to the ML model is shown in Fig. 6a left panel. For the same dataset, the energy spread is chosen as the only training output variable (easily expandable to include both energy and energy spread). We applied PCT in four different cases, corresponding to the ones with both x and y jitters (case I), with x jitter only (case II), with y jitter only (case III), and with both x and y jitters removed (case IV). The difference between the top and bottom graphs in the right column of Fig. 6a is that the top graph corresponds to case I (after application of PCT-*v*1 and with all four peaks, labeled 1, 2, 3, and 4, misaligned in the radial direction (*R*) due to the x and y jitters) and the bottom graph is for case IV (after application of PCT-*v*2 and with these four peaks are perfectly aligned, thanks to the removal of the x and y jitters).

The training and validation sets are used to fit the model and to prevent the model from overfitting, while the test set is used to check the accuracy of the trained model. For the 200 untrained images from the test set, the MSEs of case- I, II, III, and IV are plotted in Fig. 6b. The corresponding MSE values are 0.0492, 0.0183, 0.0237, and 0.0092, respectively. Training losses of the energy spread for the four cases are plotted in Fig. 6c as the light blue, light orange, dark orange, and dark blue curves, respectively. Thanks to the removal of x and y jitters, the generalization error in case IV is more than 5 times smaller than in case I. As expected, cases II and III have similar errors, which are between the errors of cases I and IV. Thus, we demonstrated that PCT-*v*2 has a more than a factor of five reduction in the MSE due to the removal of x and y jitters. By repositioning each BD pattern to the center of the corresponding image, we expect that the fidelity of the energy spread prediction via PCT-*v*2 could be immune from the impact of x and y jitters.

### BPA for improving the divergence prediction

For the prediction of the divergence and the spatial position of an electron beam, we select a rectangular section in every BD pattern, which covers the brightest Bragg peak with the minimum area. The criterion for determining the size of the selected area for BPA is that the section must cover $\pm 5 \cdot \sigma_{x,y}$ (Bragg peak widths in x and y directions), even when the electron beam has the maximum jitters in both x and y directions. This section is only used for the analysis of x and y divergences and spatial positions. The characteristic of the divergence is that it broadens each BD peak similarly in a BD pattern in the same direction as the divergence. In contrast, the feature of the spatial position jitter is to transversely shift the image on the detector with the same amount of the spatial position jitter. Therefore, the selected section with the brightest BD peak could be used to train an ML model, which only predicts the x and y divergences and spatial positions of the electron beam. The reduction of the image size and the selection of the peak with the highest intensity could significantly improve the speed as well as guarantee high fidelity of the prediction. Also, it is important that the availability of x and y spatial positions allows the removal of x and y position jitters from each BD pattern, which is required as the first step of PCT-v2.

**Figure 7:**
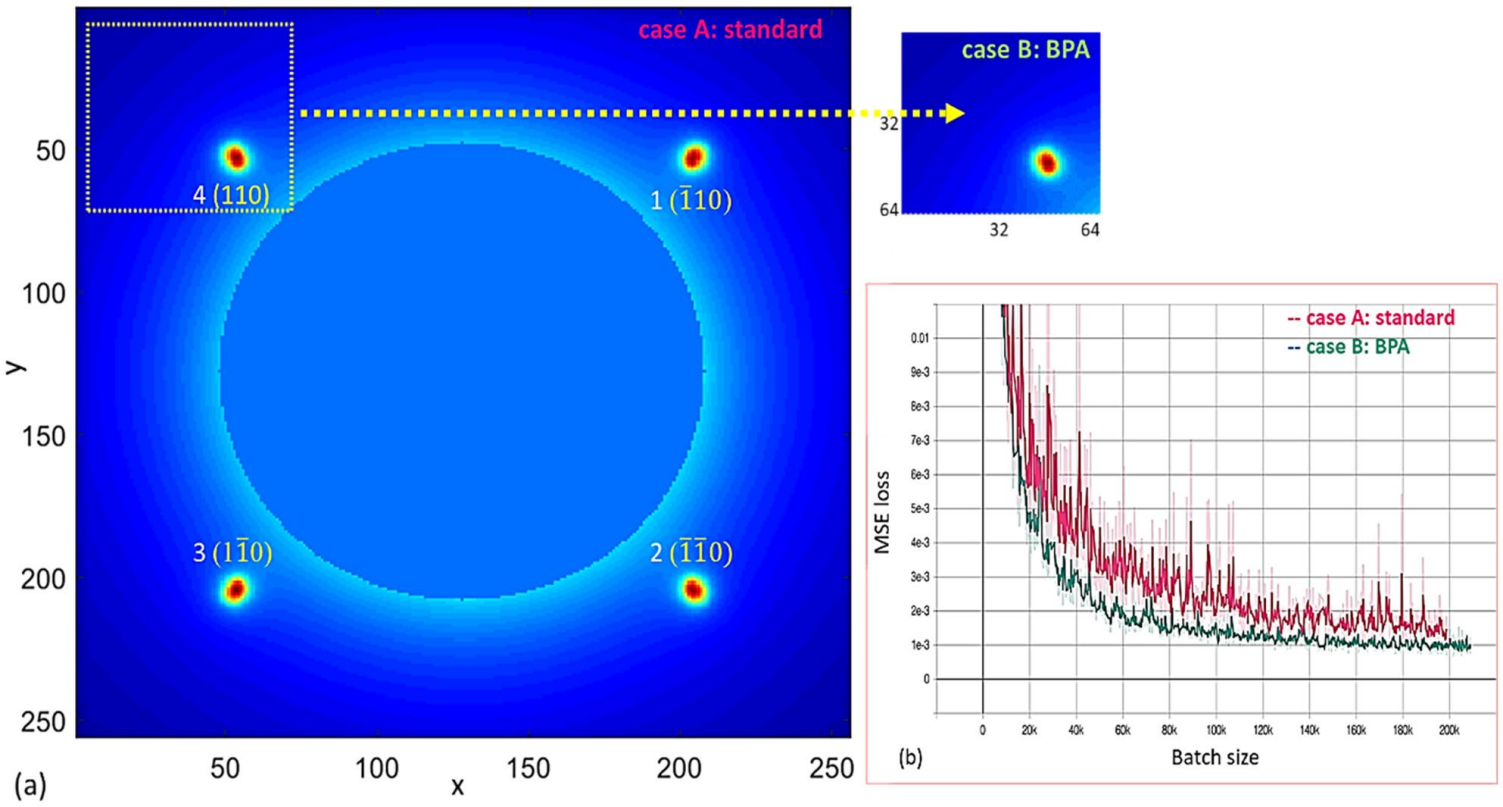
(**a**) An example of simulated BD patterns of SrTiO_3_ single crystal along [001] direction in the dataset with the RMS energy spread of (1.3·10^–2^) is plotted as the left graph. The image has a size of 256·256 pixels, with an angular resolution of 80 pixel/mrad in the EDP simulation. The x and y spatial pointing jitters in RMS are 36 μrad. For each image, the view angles are the same in *x* and *y* directions: ± 1.6 mrad. Miller indexes of those four selected Bragg peaks, 1, 2, 3, and 4, are ($\overline{1}10$), ($\overline{1}\overline{1}0)$, (1 $\overline{1}$ 0), and (110), respectively. The BD peak highlighted as the yellow dotted square with the size of 64·64 pixels is selected for the BPA training, as shown in right plot of Fig. 7a. (**b**) Training loss vs batch size is plotted as the red and green curves regarding cases A and B, respectively. The smoothed training losses are plotted on top of the raw data (the translucent curves).

We use the same set of ten thousand images. One example of the input to the ML model is shown in the left panel of Fig. 7a. The x and y divergences and spatial positions are chosen as the four training variables. We performed two types of trainings: (1) case A (standard)—for each image, the entire BD pattern ($256 \cdot 256$ pixels) is used for the training; (2) case B (BPA) – for every image, only the area covering the brightness BD peak ($64 \cdot 64$ pixels) is selected for the training, highlighted as the yellow dotted square in the left graph and also shown as the small graph in Fig. 7a top right. The average training losses of all four variables (x and y divergences and positions) are plotted in Fig. 7b as the red and green curves for case A (standard) and case B (BPA), respectively. To make a comparison between cases A and B, the MSEs regarding the test dataset for x and y jitters and divergences, including before (columns with the label “MSE”) and after (columns with the label of “unit converted MSE”) the unit conversion, and the time taken for the training are listed in Table 3. As expected, case B performs better than case A, with a 15% reduction in MSEs and two times faster in speed, since the selected highest intensity peak with the maximum signal-to-noise ratio contains all information required to predict the x and y divergences and spatial positions.

**Table 3:** MSEs for the electron beam properties—x and y jitters and divergences (p_1_, p_2_, p_3_, p_4_), including before and after the unit conversion, are listed for two cases A (standard) and B (BPA).

Beam properties	Formula	Description	Unit	Case A: standard		Case B: BPA	
				MSE	Unit converted MSE	MSE	Unit converted MSE
*p*_*1*_	*avg(x)*	x-position	μm	0.0021	0.1087	0.0020	0.1048
*p*_*2*_	*avg(y)*	y-position	μm	0.0018	0.0940	0.0018	0.0940
*p*_*3*_	*std(x')*	x-divergence	μrad	0.0320	0.3200	0.0289	0.2890
*p*_*4*_	*std(y')*	y-divergence	μrad	0.0400	0.4000	0.0323	0.3230
*Time*	*T*	Training time	min	30		14	

The times taken for the training process are 30 min and 14 min for case A and B, respectively.

## Method

The start-to-end simulations are performed through particle tracking via GPT from the gun to the sample and the EDP simulation of a wave-like electron being diffracted by the sample. A two-stage approach is used to build the ML models, GTS-ML from the gun to the sample and STD-ML from the sample to the detector. The BD patterns as the input and the electron beam properties as the data label are applied to train the STD-ML model. To automate the UED instrument, the machine parameters and the electron beam properties as the data label and input data, respectively, are applied to build the GTS-ML model.

To improve the STD-ML model, we decouple the unique features of the beam energy spread and divergence via PCT-*v*2 and BPA, which preserve the radial symmetry and x–y translation invariant, respectively. The BPA method is applied to build an ML model that predicts the x and y divergences as well as spatial position jitters. Removing the x and y position jitters makes each BD pattern to be centered in the image, hence, to be ready for PCT-*v*2. Then, those PCT BD patterns are used to train an ML model, which only predicts the energy spread and energy. As a result, the precisions of the energy spread and divergence predictions are improved significantly.

In practice, a training set consists of the measurements of BD patterns from a sample under different machine parameters. The previously trained STD-ML model is used to determine the beam properties at the sample, which in turn, is used to train the GTS-ML model. To use the ML model in an experiment, the users only need to provide the desired beam properties at the sample; with the real-time BD pattern recorded by the detector, the STD-ML model outputs the beam properties at the sample, and, by comparing the model predicted and user targeted beam properties, the GTS-ML model predicts the required machine parameters.

## Discussion

The concept demonstrated in simulation in this study is directly applicable to automate the UED instrument in experiments. The proposed procedures are: set the machine parameters of the accelerator based on the table (10,000·6). Each set of the 6 machine parameters determines a BD pattern on the detector. We record all BD patterns. Using those BD patterns as input, we apply the pretrained STD-ML model to predict the electron beam properties at the sample, which are used as the input to the GTS-ML model as well as to provide real time diagnostic information. Subsequently, we use the machine parameters and the electron beam properties as the data label and the input data of the training dataset, respectively, to build the GTS-ML model.

A proof-of-principle simulation study has been done via the two-stage ML model. It is evident that the precision and speed of the prediction are adequate for fully automated UED operation. In the experimental case, the entire process, including setting up the UED instrument, recording BD patterns, loading the dataset to the computer, training the ML model, and updating the model in the operating system of the UED instrument, will take just a few hours (e.g., 4 h in collecting data and 10 min in training for the 10 k-data case on an RTX2080 GPU). The estimation assumes that the ML model has been established prior to the experiment and the time is only spent in updating the model. Since the GTS-ML model is sample independent, the model needs to be updated less often (e.g., once in a few weeks); it is performed only when there is a change of the machine calibration, or the commissioning of a new operational mode. Occasional retraining is needed when the beam falls out of the range on which the GTS-ML model was trained. Similarly, STD-ML needs to be updated only when there is a sample change or detector replacement.

The study demonstrates that the two-stage model enables the full automation of the UED instrument. The BPA method reduces the image size by more than a factor of 10. These images are used to train an ML model, which predicts the beam divergences and spatial position jitters with a guaranteed high fidelity. Besides, the predicted x and y position jitters are needed to center those BD patterns before the application of PCT-v2. Then, the PCT BD patterns are used to train an ML model, which only predicts the energy spread and energy and hence has a significantly improved precision (> 5). Furthermore, compared to the proposed method in our earlier study^1^, the global optimization enabled by the ML via Latin hypercube sampling scheme^33^ provides a full coverage of the parameter space of a UED instrument, and as a result, allows more electrons to be packed (with 150 times brighter) in the most important dimensions, i.e., divergences and energy spread, as shown in Table 4. We provide a roadmap toward the full automation of the UED operation and real-time diagnosis of electron beam properties with a comprehensive set of beam parameters. The two-stage approach can be easily extended to other types of accelerator facilities. Eliminating the setup time for a UED experiment and providing the ultrahigh bright electron beam to the UED/UEM with a single-shot imaging capability can be a game changer in the ultrafast science and technology.

**Table 4:** The operational and ML optimized electron beam properties at the standard charge of 0.5 pC are shown as the left and right columns, respectively.

	Conventional e- beam properties	ML optimized e- beam properties
x-divergence (µrad)	140	18
y-divergence (µrad)	140	18
Normalized energy spread (%)	0.025	0.01
Improvement factor		151

We only consider the x and y divergences and normalize energy spread of the electron beam, which ultimately limit the UED/UEM instrumental resolution.

## Data Availability

The datasets generated and analyzed during the current study are not publicly available due to the reason that we want to know who has an interest in our datasets but are available from the corresponding author on reasonable request.
